# Thermal Transport and Thermoelectric Effect in Composites of Alumina and Graphene-Augmented Alumina Nanofibers

**DOI:** 10.3390/ma14092242

**Published:** 2021-04-27

**Authors:** Ali Saffar Shamshirgar, Manuel Belmonte, Girish C. Tewari, Rocío E. Rojas Hernández, Jani Seitsonen, Roman Ivanov, Maarit Karppinen, Pilar Miranzo, Irina Hussainova

**Affiliations:** 1 Department of Mechanical and Industrial Engineering, Tallinn University of Technology, 19086 Tallinn, Estonia; rocio.rojas@taltech.ee (R.E.R.H.); roman.ivanov@taltech.ee (R.I.); 2 Institute of Ceramics and Glass (ICV-CSIC), Kelsen 5, 28049 Madrid, Spain; mbelmonte@icv.csic.es (M.B.); pmiranzo@icv.csic.es (P.M.); 3 Department of Chemistry and Materials Science, Aalto University, FI-00076 Aalto, Finland; girish.tewari@aalto.fi (G.C.T.); maarit.karppinen@aalto.fi (M.K.); 4 Department of Applied Physics, Aalto University, FI-00076 Aalto, Finland; jani.seitsonen@aalto.fi

**Keywords:** nanofibers, graphene, ceramic, thermal conductivity, thermoelectric

## Abstract

The remarkable tunability of 2D carbon structures combined with their non-toxicity renders them interesting candidates for thermoelectric applications. Despite some limitations related to their high thermal conductivity and low Seebeck coefficients, several other unique properties of the graphene-like structures could out-weight these weaknesses in some applications. In this study, hybrid structures of alumina ceramics and graphene encapsulated alumina nanofibers are processed by spark plasma sintering to exploit advantages of thermoelectric properties of graphene and high stiffness of alumina. The paper focuses on thermal and electronic transport properties of the systems with varying content of nanofillers (1–25 wt.%) and demonstrates an increase of the Seebeck coefficient and a reduction of the thermal conductivity with an increase in filler content. As a result, the highest thermoelectric figure of merit is achieved in a sample with 25 wt.% of the fillers corresponding to ~3 wt.% of graphene content. The graphene encapsulated nanofibrous fillers, thus, show promising potential for thermoelectric material designs by tuning their properties via carrier density modification and Fermi engineering through doping.

## 1. Introduction

Almost all energy conversion systems are associated with waste of energy in the form of heat. Thermoelectric (TE) conversion is an attractive and technologically viable solution to directly harvest this waste heat. The thermoelectric effect is described as the conversion of heat (temperature gradient) to a voltage potential through the Seebeck effect and vice versa through the Peltier effect. The efficiency of a TE material is defined by the thermoelectric figure-of-merit which is proportional to the Seebeck coefficient and electrical conductivity of the material, and inversely to its thermal conductivity. Therefore, in order to achieve high conversion efficiency, the TE materials are required to have a high Seebeck coefficient, high electrical conductivity, and low thermal conductivity. However, the tradeoff that exists between these three properties makes the design of TE materials challenging. As an example, electrical conductivity increases by increasing carrier density through the following relationship σ = neμ. However, an increase in carrier density results in a decline in Seebeck coefficient. An increase of the carrier density also increases the electrical contribution to the thermal conductivity and subsequently a decline in the thermoelectric Figure of Merit [1]. A number of organic [2] and inorganic [3] materials have been exploited for thermoelectric applications. However, many of the applicable semiconductor materials including but not limited to Bi_2_Te_3_ [4], PbTe [5], and Sb_2_Te_3_ [6] are cost-intensive to manufacture and constitute of rare and/or toxic elements [1,6]. Carbon-based structures have attracted a great deal of attention due to their abundance, nontoxicity, and ease of processing [7,8,9,10,11,12,13,14,15]. Among them, graphene presents a remarkable potential for precise nanostructure tailoring and subsequently, on-demand tuning of properties [16]. Introducing bandgap to graphene through doping, controlling carrier density, and Fermi engineering have a direct effect on the Seebeck coefficient. Furthermore, embedding graphene into nanostructures can provide a large number of boundaries, which strongly scatter phonons, resulting in significantly suppressed thermal conductivity [17,18].

Recently, hybrid nanofillers representing graphene-augmented alumina nanofibers (GAIN) were produced through a one-step catalyst-free chemical vapor deposition (CVD) and used as nanofillers to manufacture GAIN/α-Al_2_O_3_ composites with various graphene contents. The results have shown that the presence of GAIN fillers up to 10 wt.% does not influence hardness, however, a significant inhibition of the grain growth was observed. It was also shown that the electrical percolation threshold of the composites lies at 3 wt.% filler content [19]. In the current study, the GAIN/α-Al_2_O_3_ structures are consolidated using spark plasma sintering (SPS) and their TE properties are characterized. Moreover, an in-depth discussion on thermal conductivity and its anisotropy in the structures is provided.

## 2. Experimental

### 2.1. Processing of the Composites

As the matrix, commercially available α-alumina nano-powder with an average particle size of 100 nm (TM-DAR, Taimei, Tokyo, Japan) was used. As nanofillers, GAIN fibers produced with the help of a cost-effective catalyst-free CVD method of carbon deposition onto ceramic nanostructures as detailed in [20,21] were considered. GAIN fibers were crushed in a mortar, dispersed in ethanol, and sonicated using a sonication rod (Hielscher UP400S, Hielscher Ultrasonics, Teltow, Germany) for 20 min at 30 W using the alternative regime of 4 s ON—1 s OFF, and dried in a muffle furnace following the procedure detailed in [22]. The dried fibers of 400–800 nm in length and 8–20 nm in diameter, were weighed with corresponding amounts of α-alumina, in order to prepare samples with 1–25 wt.% GAIN. Each powder composition was further sonicated for 20 min at 30 W for homogenization. After drying at 70 °C for 12 h, the blends were collected, sieved, and sintered using spark plasma sintering (FCT System GmbH SPS furnace, FCT System GmbH, Frankenblick, Germany) in a graphite die with 20 mm diameter at 1150 °C and 75 MPa in a vacuum. A full index of the samples is listed in Table 1.

### 2.2. Characterization

The microstructural characterization was carried out using scanning electron microscope (HR-SEM Zeiss Merlin, ZEISS, Oberkochen, Germany) equipped with an energy dispersive X-ray spectrometer (Bruker EDX-XFlash6/30 detector, Bruker, Billerica, MA, USA) with an operating voltage of 5 kV. The morphology of the hybrid GAIN was examined by JEOL JEM-2800 high resolution transmission electron microscope (HRTEM, JEOL Ltd., Tokyo, Japan). Confocal Raman spectra of the synthesized materials were recorded using a Horiba’s LabRAM 800 (Horiba, Ltd., Kyoto, Japan) high-resolution spectrometer equipped with a 532 nm laser excitation wavelength at room temperature (RT) and a 50× objective lens (NA = 0.95). Collected spectra are processed and analyzed using Witec Control Plus software 2.08. The incident laser power was 2.8 mW. Carbon content of the GAIN fillers was determined using a LECO CS 200 carbon-Sulphur analyzer (LECO, Minato, Japan) based on arithmetic mean of three readings. The densities (*ρ*) of the sintered specimens were determined using the Archimedes method with distilled water as the immersive media. The rule of mixtures using the manufacturers’ density specification for alumina powder (3.96 g·cm^−3^), alumina fibers (3.65 g·cm^−3^), and published density value for graphene (2.2 g·cm^−3^) was used to calculate the relative densities. The in-plane thermal diffusivity (*α^||^*) (perpendicular to the SPS pressure axis) was measured on discs with 20 mm diameter and 600 µm thickness by the laser-flash method (Thermaflash 2200, Holometrix, Netzsch GmbH, Selb, Germany). The through-plane thermal diffusivity (*α*^⊥^) (Transversal to the SPS pressure axis) was measured on square samples (8.8 × 8.8 mm^2^) from 298 to 1073 K. Prior to the procedure, thin matte graphite layers were sprayed onto the samples’ surfaces exposed to the laser excitation; to avoid energy loss due to surface reflection and maximize absorption. The low-temperature thermoelectric transport properties were measured using a physical property measurement system (PPMS, Quantum Design GmbH, San Diego, CA, USA); equipped with 9 T magnetic field). A set of specimens were cut using a precision diamond wheel to bars of 5 × 10 × 1 mm^3^. Simultaneously, resistivity (*ρ*), Seebeck coefficient (*S*), and thermal conductivity were measured using the thermal transport puck (TTO) of the PPMS on the rectangular bars with the four-probe contact arrangement. In this procedure, at the steady state, a small amount of heat is applied to one end of the rectangular bars and the temperature difference together with the Seebeck voltage are simultaneously recorded. The Seebeck coefficient is estimated by dividing the Seebeck voltage by the temperature difference. The thermal conductivity (*k^||^*) is calculated using the dimensions of the specimen, distance between the probes, and the temperature difference.

## 3. Results and Discussion

### 3.1. Microstructure and Composition

**Figure 1:**
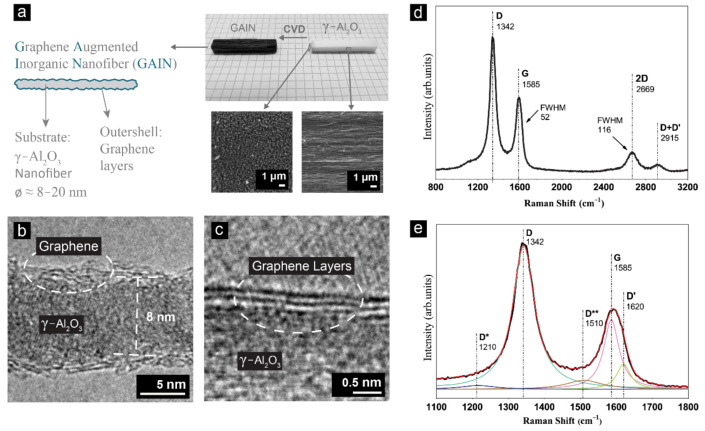
(**a**) Schematic of GAIN with γ-Al_2_O_3_ core and polycrystalline graphene coating together with SEM images of γ-Al_2_O_3_ nanofibers; (**b**,**c**) HRTEM images of a GAIN fiber with 2–3 layers of graphene coating; (**d**) Raman spectrum of GAIN; and (**e**) deconvolution of the peaks providing details on the Raman shift ranging from 1100 to 1800 cm^−1^.

A schematic view of GAIN fillers and the optical images of γ-Al_2_O_3_ nanofibers before and after CVD are provided in Figure 1a. SEM images in Figure 1a demonstrate self-alignment of alumina nanofibers. In Figure 1b, a discontinues highly defective multilayered graphene coating is observed. TEM images of various regions of the fibers indicate a variety of stacked graphene structures ranging from 2 to 5 layers with a wide range of layer lengths. An example of a tri-layer graphene structure on the γ-Al_2_O_3_ fibers is shown in Figure 1c.

Raman spectra presented in Figure 1d,e show the main features of carbon materials, Raman D, G, and 2D peaks. The high intensity of the D peak as compared to G, indicates very small sp^2^ graphene domains which is consistent with the HRTEM observations. Based on the double resonant Raman scattering theory, the I_D_/I_G_ ratio of ~1.8 for GAIN corresponds to on-site and vacancy-like defect types [23,24]. The 2D peak for single-layer graphene consists of a single component that is ~5 times stronger than the G peak intensity. The broadening of the 2D peak is directly in connection with increasing graphene layer count, and its shape and locations can be used to monitor layer count and carbon structure type [25]. In GAIN structures the full width at half maximum (FWHM) of the 2D peak centered at 2669 cm^−1^ is ~116, almost five times that of the 2D peak of a common single-layer graphene, which together with the presence of a notable first order D peak at 1342 cm^−1^ corresponds to the results reported for turbostratic carbon [25]. Functionalization of graphene with hydrogen and sp^3^ hybridization introduce a peak at ~2915 cm^−1^. This peak is the second order of the intra-valley D′ peak at ~1620 [25], which is present in defected carbon structures [26]. The 2D band is 21 cm^−1^ downshifted as a result of the local strain caused by the γ-Al_2_O_3_ substrate [27]. In other words, a lattice mismatch between the substrate and graphene exerts physical strain on the graphene and consequently decreases phonon energies, promoting a downshift. In the case of a uniaxial strain, the G peak is split into two features, where the second one (D′) at ~1620 cm^−1^ is in connection with the D peak [28]. Considering that D and 2D peak positions in carbon are dispersive, for comparison of the Raman shifts the laser excitation energy needs to be taken into account [25], hence, the cited reference positions are reported values obtained by 532 nm laser. In addition to the 2D band, both mechanical strain and increasing number of graphene layers should promote a downshift of the G band; however, for the GAIN fillers, the G peak is 5 cm^−1^ upshifted as compared to the 1580 cm^−1^ for single layer free-standing graphene [29]. This observation can be attributed to the doping effect of the substrate γ-Al_2_O_3_ [30]. To clarify, the Raman G peak corresponds to the in-plane vibration of the E_2g_ phonons at the Γ point at the Brillouin zone center. Both *n-* and *p*-doping shift Fermi level away from the Diract point, decreasing the probability of charge carriers’ recombination. This results in non-adiabatic perturbation of the phonons, removing the Kohn anomaly. The outcome is an increase in the phonon energy of the G peak, and the corresponding upshift [31,32]. Deconvolution of the 1100–1800 cm^−1^ range (Figure 1e) shows that in addition to the D′, two more defect-driven D* and D** peaks are present at 1210 and 1510 cm^−1^, respectively. The D* is known to be in connection with sp^3^ orbitals in disordered amorphous carbon [33] and nanocrystalline diamond [34]. The D* was also observed in graphene oxide where vibration restrictions were posed by the presence of oxygen-containing groups [35] and few-layer wrinkled graphene (FLwG) [36]. However, both bands were postulated by Ferarri et al. [34] to be in connection with sp^2^-bonded configurations of transpolyacetylene segments at grain boundaries and surfaces of CVD carbon.

**Figure 2:**
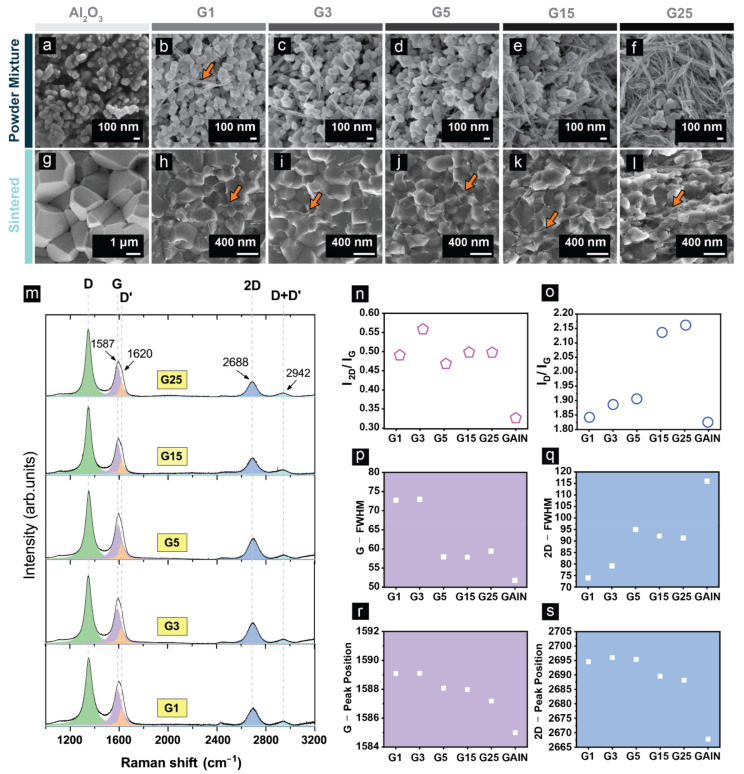
SEM micrographs of (**a**–**f**) alumina powder with various GAIN content; (**g**–**l**) sintered bulks consisting of reference alumina and GAIN/alumina compositions; (**m**) Raman spectra of composites with 1–25 wt.% GAIN content; (**n**) I_2D_/I_G_ ratio; (**o**) I_D_/I_G_ ratio; (**p**) G peak FWHM; (**q**) 2D peak FWHM; (**r**) G peak positions; (**s**) 2D peak position for GAIN and composites containing 1–25 wt.% GAIN fillers.

In Figure 2a–f the dispersion of the GAIN fillers in the powder mixtures is demonstrated in the SEM micrographs. SEM images and the microstructural features of the SPS processed composites are shown in Figure 2g–l. A gradual decrease in the grain size with an increase in the filler content is well-recognized starting from coarse 1–5 µm grains in the pristine alumina (Figure 2g) to the refined grains of around 100 nm in the G25 composite with 25 wt.% GAIN (Figure 2l). At the filler contents higher than 5 wt.%, the relative density rapidly decreases due to the pinning effect and reduced mass transport in the presence of high carbon content, which hinders densification [37]. The presence of γ-Al_2_O_3_ as the core of the GAIN in the α-Al_2_O_3_ matrix can trigger γ → δ → θ → α-Al_2_O_3_ phase transformation process in the temperature interval of 1050–1200 °C producing a vermicular microstructure [38,39]. However, under the employed sintering parameters, none of the samples demonstrate features of a vermicular structure. A discussion on the sintering temperature and its effect on the microstructure of GAIN/Al_2_O_3_ is detailed in [40]. The GAIN fillers are visibly located at the grain boundaries with a slight preferential orientation which is more pronounced for the composites of the high filler contents. A discussion on anisotropy will be provided in Section 3.2.

Even though the structure of the graphene is preserved in the sintering procedure, the upshift of the G and 2D peaks and broadening of FWHM of G peak in comparison with GAIN, can be evidence of both *n-* and *p*-doping induced by the α-Al_2_O_3_ matrix (Figure 2m–s) [25]. However, the presence of aluminum interstitials and oxygen vacancies in the α-Al_2_O_3_ is more likely to contribute to the localization of electrons in the graphene sheets, promoting *p*-doping. Specifically, the upshift of the 2D band in composite structures (Figure 2s) as compared to GAIN fibers can be another supporting argument for the *p*-doping induced by the matrix [41]. A further evidence of this interpretation is the positive sign of the Seebeck coefficient that will be discussed in Section 3.3. Regardless of the doping effect in the composites, the presence of compressive stresses emanated from the opposite thermal expansion and contraction of the carbon and alumina can increase the phonon frequency of various bands and virtually cause an upshift of the spectrum [27] as evidenced in Figure 2r,s.

### 3.2. Thermal Properties

**Figure 3:**
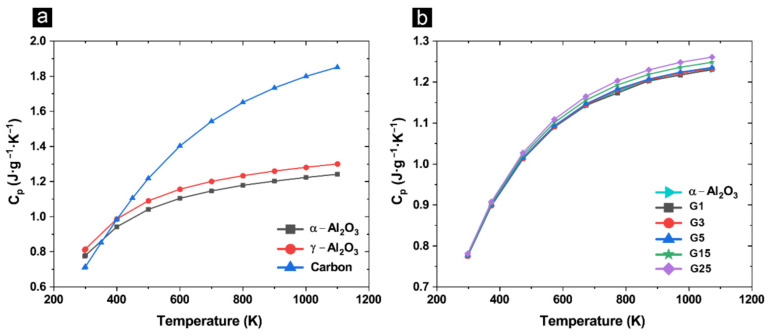
(**a**) Specific heat capacity (C_p_) versus temperature for alumina (α and γ) and carbon using NIST-JANAF database; and (**b**) calculated C_p_ of the composites as a function of the GAIN content and temperature.

To calculate thermal conductivity (*k*) using the following equation *k = α·ρ·C_p_*, values for specific heat capacity (*C_p_*) of γ-Al_2_O_3_, α-Al_2_O_3_, and carbon were extracted from the NIST-JANAF database [42] (Figure 3a) and the corresponding *C_p_* values of the specimens were determined using the rule of mixture. The density (*ρ*) values were obtained from Archimedes density measurement and diffusivity (*α*) from the laser flash method. The *C_p_* values of the composites are plotted as a function of temperature in Figure 3b.

**Figure 4:**
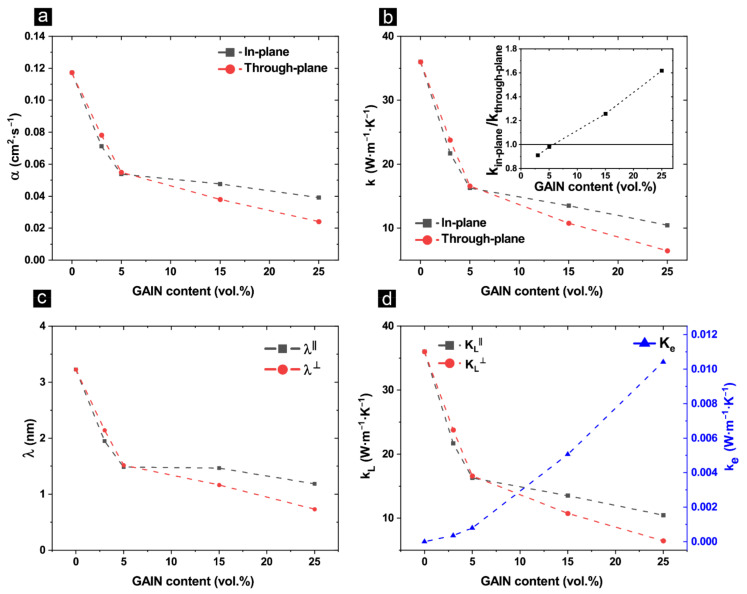
(**a**) Thermal diffusivity and (**b**) thermal conductivity as a function of GAIN content—inset: in-plane vs. through-plane thermal conductivity; (**c**) in-plane and through-plane mean free path as a function of GAIN content; (**d**) lattice thermal conductivity (K_L_) and electronic thermal conductivity (K_e_) as a function of GAIN content.

Both in-plane and through-plane thermal diffusivity and conductivity decrease as a function of GAIN content, as seen in Figure 4a,b. At filler contents higher than 5 wt.%, the through-plane values for both diffusivity and conductivity decrease at a higher rate as compared to in-plane values indicating a certain anisotropy in the microstructure and/or the alignment of the fillers perpendicular to the SPS pressure axis. The impact of this anisotropy can be seen in Figure 4b-inset, where the in-plane to through-plane ratio is plotted as a function of GAIN content. The higher in-plane thermal conductivity was also reported for SWCNT/alumina composites with 10 and 15 vol. % CNT content [43]. For both *α* and *k*, a significant drop can be observed specifically at up to 5 wt.% filler content, indicating a large contribution of the thermal contact and boundary resistance introduced by the presence of the fillers. Moreover, phonon-defect scattering at *Al* interstitials and *O* vacancies and presumed *p*-doping are other possible limiting factors for the thermal transport. Therefore, a detailed analysis is required to understand the thermal conductivity of the GAIN/alumina composites.

Employing Wiedemann–Franz law, the electronic (*K_e_*) contribution to the total thermal conductivity (*k*) was calculated using Equation (1) in which *L* is the Lorenz constant equal to 2.44 × 10^−8^ W*·*Ω*·*K^−2^, *σ* is electrical conductivity and *T* is the room temperature [44]. The corresponding values for composite specimens were calculated and subtracted from the total transport to obtain lattice thermal conductivity (*K_L_*). The *K_L_* can be correlated to phonon mean free path (*λ*) using Equation (2), in which *C_p_* is the specific heat capacity, and *V* is the phonon group velocity of the bulk material [45]. The phonon group velocity of the bulk can be determined using Equation (3), where *ρ* is the bulk density, and *E* is the elastic modulus, attained from the previous paper of the authors [40]. The values of *λ* are presented in Figure 4c for both in-plane and through-plane directions. The estimated value of *λ* for α-alumina at 3.2 nm agrees with the reported value for polycrystalline bulks [46] and decreases by increasing GAIN content. Since electrical conductivity in all composites is in the semiconducting range, the contribution of the electronic component to the thermal conductivity is negligible (Figure 4d), therefore, the main mode of thermal transport is expected to be by phonon interactions. As the GAIN fillers are added into the alumina matrix, impurity scattering, and boundary scattering interactions limit the mean free path which in turn negatively impacts diffusivity. The latter interaction is presumed to be the reason for lower *λ*^⊥^*,* since theoretically, thermal boundary resistance is more significant in transversal direction to filler alignment (refer to Section 3.1 for the discussion on microstructural anisotropy at high filler content). At filler contents higher than 5 wt.%, despite the decrease in relative density, a slower decline of the α and k is associated with the connectivity of the fillers preserving phonon mean free path specifically for in-plane direction (Figure 4a–c) [18]. Additionally, since the grain size distribution is higher at low filler contents and decreases as a function of GAIN, the contribution of the microstructure to the phonon mean free path should not be neglected. In the present case, since the values of a few nanometers for phonon mean free path are much smaller than the grain sizes of the composites (several hundreds of nanometers), defect and impurity scatterings could be contributing more to the decrease of the phonon mean free path as compared to the boundary scattering [47]. Similar observation was done on graphene–alumina composites [48] and composites of MWCNT/alumina where the 35 W*·*m^−1^*·*K^−1^ room temperature thermal conductivity of alumina matrix decreased as a function of MWCNT to 15 W*·*m^−1^*·*K^−1^ for ~10 vol.% MWCNT [49].

K_e_ = L *×* σ *×* T (1)

(2) $$K_{L}=\frac{1}{3}C_{p}\times v\times \lambda$$

(3) $$v=\sqrt{E}\times \frac{1}{\sqrt{\rho }}$$

**Figure 5:**
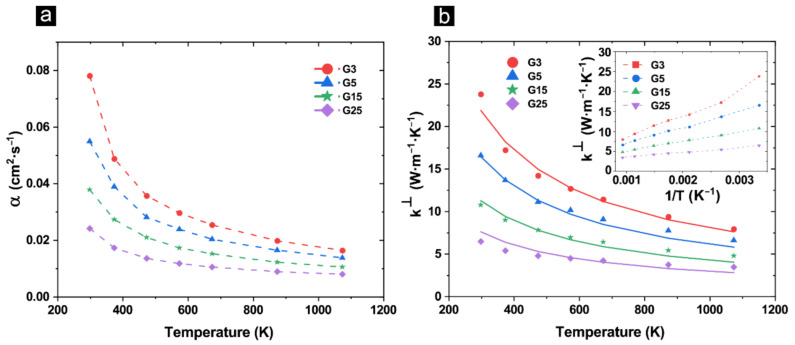
(**a**) through-plane diffusivity of the composite structures as a function of temperature; (**b**) through-plane thermal conductivity of the composite structures as a function of temperature (solid lines represent fittings for k~T^-B^)—inset: effective thermal conductivity versus 1/T.

In all compositions, *α*^⊥^ and *k*^⊥^ decrease by increasing temperature from 293 K to 1100 K (Figure 5a,b). Figure 5b-inset shows the *k*^⊥^ versus reciprocal of temperature for the composite structures. Theoretically, a straight line in this plot is an artifact of anharmonic Umklapp phonon scattering. However, in all composites, a slight deviation from a liner relationship, suggests presence of extra phonon scattering mechanisms. It is known that the presence of GAIN at grain boundaries of alumina matrix can induce interface scattering (thermal boundary resistance) which reduce mean free path at room temperature. Nonetheless, since the boundary resistance decreases by temperature, its contribution to scattering processes are less pronounced at elevated temperature [50]. In fact, temperature dependency of the GAIN/alumina composites follow the k~T^-B^ relation where the exponent “*B*” varies from 0.82 to 0.78 for G3 to G25 respectively (predicted k~T^-B^ values are shown as solid lines in Figure 5b).

### 3.3. Thermoelectric Performance

**Figure 6:**
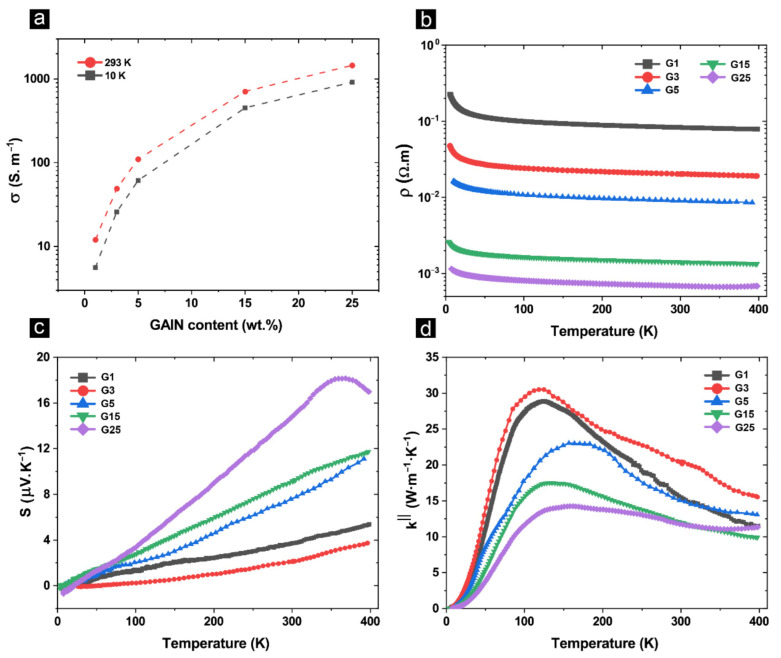
(**a**) electrical conductivity as a function of GAIN content; (**b**) Temperature dependence of resistivity; (**c**) temperature dependence of Seebeck coefficient; (**d**) low temperature in-plane thermal conductivity of the composite structures.

Resistivity decreases as a function of GAIN content (Figure 6a) and decreases in all samples by increasing temperature from 4 K to 400 K (Figure 6b). The slope of the *ρ* vs. *T* shows the semiconducting/semi-metallic nature of the transport while the semi-metallic behavior is more pronounced at higher filler contents. The bandgap was calculated from the slope of the Arrhenius plot for bulk composites with 1–25 wt.% GAIN and ranges from 1.0 to 0.6 meV. Previously it was shown that both carrier types are present in the GAIN/alumina structures owing to the *n*-type GAIN fillers and the *p*-doping of the matrix alumina in the presence of point defects such as *Al* interstitials and *O* vacancies [40]. The positive values of the Seebeck coefficient in Figure 6c prove that the majority carriers are *p*-type. The values of the Seebeck coefficient increase both with temperature and GAIN content. In fact, induced Seebeck voltage is directly in connection with the diffusion process of the charged species and phonon drag. In graphene structures, the magnitude and sign of the Seebeck coefficient are influenced by the asymmetry in the distribution of the density of states near the Fermi level [2,44]. In general, increasing the Seebeck coefficient is inversely proportional to carrier density. However, increasing mobility and decreasing bandgap are associated with augmenting both *σ* and *S* [1]. Increasing carrier mobility and carrier density was established as a function of GAIN content [40]. Therefore, in the sample with the highest GAIN content (25 wt.%), and correspondingly higher carrier density and mobility, an overall higher *S* is achieved which peaks at 18 µ*·*V*·*K^−1^ at 350 K. Similar values were obtained for 17 vol.% GNP-Si_3_N_4_ composites [51]. Thermal conductivity rapidly increases from 4 K to peak at 120 K due to a rapid increase in thermally enabled vibration modes (Figure 6d). As the temperature increases, impurity scattering, defect-phonon scattering, and phonon-electron scattering together with the promotion of more charge carriers result in a decline of *k^||^* up to 400 K [44]. Thermal conductivity also decreases as a function of GAIN content due to a decline in the relative density and impurity scattering as a higher concentration of the fillers are introduced, following the same mechanisms discussed in Section 3.2. The thermal conductivity values at room temperature measured by PPMS, agree with the calculated values based on diffusivity measurement using the laser flash method.

**Figure 7:**
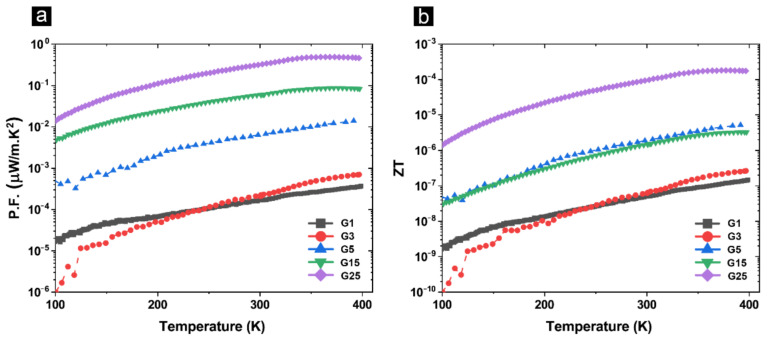
(**a**) Temperature dependence of power factor and (**b**) thermoelectric figure-of-merit, of composite structures.

The power factor (P.F.) and ZT values were calculated using Equations (4) and (5), respectively, and their corresponding temperature dependence values are presented in Figure 7a,b. In these equations, *σ* is electrical conductivity, S is the Seebeck coefficient, k is the thermal conductivity and T is temperature.

P.F. = σ S^2^ (4)

(5) $$\mathrm{ZT}=\frac{{\sigma S}^{2}T}{k}$$

The highest P.F. is expectedly achieved in the sample G25 with the highest GAIN content and subsequently the highest *σ* and *S*. In fact, P.F. obeys the same pattern for *σ* and *S* for all composites, however ZT values of the samples with 5 and 15 wt.% are almost identical, suggesting the same TE efficiency attributed to comparable *S^2^* component between the two. All in all, the highest ZT is achieved in the sample with the highest filler content due to a low thermal conductivity together with the highest power factor.

## 4. Conclusions

Hybrid composites of α-alumina matrix and graphene augmented γ-Al_2_O_3_ nanofibers as fillers have been manufactured by SPS at 1150 °C under 75 MPa uniaxial pressure in a vacuum. The filler content has been varied from 1 up to 25 wt.%, which corresponds to 0.21 to 5.10 vol.% of graphene content in the form of polycrystalline graphene consisting of 2–5 layers of highly defective structures. Raman spectroscopy on GAIN fibers have shown that the 2D peak is 21 cm^−1^ downshifted attributed to the physical strain exerted on graphene by γ-Al_2_O_3_ substrate, and the G peak is 5 cm^−1^ upshifted as compared to free standing single layer graphene, which was attributed to the doping effect of the substrate and surface physisorbed water. In the matrix of alumina, GAIN fillers successfully inhibit an extensive grain growth during processing, resulting in fine-structured materials as a function of the filler content. Raman spectroscopy has indicated a slight upshift of the G and 2D bands, which are inversely related to the GAIN filler content, and attributed to the doping effect of the matrix alumina. At room temperature, thermal conductivity decreases from ~35 to ~10 W*·*m^−1^*·*K^−1^ and diffusivity from 0.12 to 0.04 cm^2^·S^−1^ both for in-plane and through-plane directions as a function of GAIN content. An anisotropic thermal conductivity has been revealed for composites with filler contents >5 wt.%, slightly above the percolation threshold. The diffusivity and the thermal conductivity are slightly higher for the in-plane direction (perpendicular to the SPS pressure axis). Moreover, further decline of the thermal conductivity from room temperature to 1100 °C was correlated to the presence of various scattering mechanisms and shown to obey k ~ T^-B^ relation. Resistivity was shown to decrease with temperature and filler content from 4 K to 400 K while majority carriers in all composites were holes. The highest Seebeck coefficient and the thermoelectric Figure of Merit have been exhibited by a composite with the highest filler content (25 wt.% GAIN). The graphene augmented nanofibers have demonstrated a great potential for thermoelectric applications by considering precise tuning of the carrier concentration and distribution of the density of states.

## Figures and Tables

**Table 1:** Graphene content and relative density of the produced samples.

Sample Name	α-Al_2_O_3_ [wt.%]	GAIN [wt.%]	GAIN [vol.%]	Graphene * [vol.%]	Relative Density
α-Al_2_O_3_	100	0	0	0	99.99
G1	99	1	1.13	0.21	99.32
G3	97	3	3.39	0.63	99.45
G5	95	5	5.65	1.04	98.98
G15	85	15	16.74	3.10	93.80
G25	75	25	27.56	5.10	89.51

* Calculated based on the measured 12 wt.% graphene content of GAIN.

## Data Availability

Not applicable.
